# An Essential Role of the Universal Polarity Protein, aPKCλ, on the Maintenance of Podocyte Slit Diaphragms

**DOI:** 10.1371/journal.pone.0004194

**Published:** 2009-01-14

**Authors:** Tomonori Hirose, Daisuke Satoh, Hidetake Kurihara, Chiho Kusaka, Hiroko Hirose, Kazunori Akimoto, Taiji Matsusaka, Iekuni Ichikawa, Tetsuo Noda, Shigeo Ohno

**Affiliations:** 1 Department of Molecular Biology, Yokohama City University Graduate School of Medical Science, Yokohama, Kanagawa, Japan; 2 Department of Anatomy, Juntendo University School of Medicine, Bunkyo-ku, Tokyo, Japan; 3 Institute of Medical Sciences, Tokai University School of Medicine, Isehara, Kanagawa, Japan; 4 Department of Bioethics, Tokai University School of Medicine, Isehara, Kanagawa, Japan; 5 Department of Cell Biology, Cancer Institute, Japanese Foundation for Cancer Research, Koto-ku, Tokyo, Japan; 6 Advanced Medical Research Center, Yokohama City University, Yokohama, Kanagawa, Japan; UT MD Anderson Cancer Center, United States of America

## Abstract

Glomerular visceral epithelial cells (podocytes) contain interdigitated processes that form specialized intercellular junctions, termed slit diaphragms, which provide a selective filtration barrier in the renal glomerulus. Analyses of disease-causing mutations in familial nephrotic syndromes and targeted mutagenesis in mice have revealed critical roles of several proteins in the assembly of slit diaphragms. The nephrin–podocin complex is the main constituent of slit diaphragms. However, the molecular mechanisms regulating these proteins to maintain the slit diaphragms are still largely unknown. Here, we demonstrate that the PAR3–atypical protein kinase C (aPKC)–PAR6β cell polarity proteins co-localize to the slit diaphragms with nephrin. Furthermore, selective depletion of aPKCλ in mouse podocytes results in the disassembly of slit diaphragms, a disturbance in apico-basal cell polarity, and focal segmental glomerulosclerosis (FSGS). The aPKC–PAR3 complex associates with the nephrin–podocin complex in podocytes through direct interaction between PAR3 and nephrin, and the kinase activity of aPKC is required for the appropriate distribution of nephrin and podocin in podocytes. These observations not only establish a critical function of the polarity proteins in the maintenance of slit diaphragms, but also imply their potential involvement in renal failure in FSGS.

## Introduction

Glomerular diseases remain the major cause of chronic and end-stage renal disease, and the number of patients with glomerular diseases is increasing [1], [2]. Because most glomerular diseases involve dysfunction of podocytes along with disassembly of slit diaphragms [1]–[5], it is necessary to understand the molecular basis for the maintenance of slit diaphragms. Mutations affecting several proteins in slit diaphragms, including nephrin and podocin (encoded by *NPHS1* and *NPHS2*, respectively), lead to glomerular disease owing to disruption of slit diaphragms [2], [5]–[7]. However, the molecular mechanisms to regulate these proteins are unclear.

The slit diaphragms share several features with the epithelial tight junctions. First, both structures are specialized cell–cell junctions critical for selective paracellular permeability [4], [8]–[11]. Second, both structures are formed at the border of apical and basolateral membrane domains and are reported to play a role in regulation of apico-basal cell polarity [3], [4], [10], [12]. Third, both structures are supported by common cytoplasmic proteins, including ZO-1, MAGI-1, and actin [13]–[15]. An evolutionarily conserved protein complex consisting of the serine/threonine protein kinase, aPKC, and two PDZ domain-containing scaffold proteins, PAR3 and PAR6, has been found to localize at the tight junctions and regulate the formation, and apico-basal epithelial polarity [10], [16], [17]. It has been revealed that PAR3 serves as a scaffold to associate aPKC with junctional proteins, and PAR6 regulates the kinase activity of aPKC [16], [17]. Currently, there is convincing evidence that many cell polarity events are commonly regulated by this complex [16], [17], although the functions of the aPKC–PAR complex in podocytes have not been clarified.

## Results

### The aPKC–PAR polarity proteins localize to slit diaphragms

**Figure 1:**
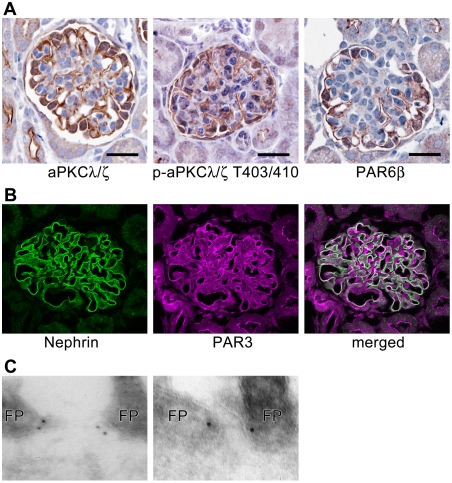
Expression and co-localization of polarity proteins to slit diaphragms. (A) In mouse kidneys at postnatal day 10 (P10), aPKCλ/ζ, phospho-aPKCλ/ζ T403/410, and PAR6β localize to the glomerular capillary tufts in a continuous pattern. Bars, 20 µm. (B) Double immunofluorescence for nephrin (green) and PAR3 (magenta). White regions show where PAR3 co-localizes with nephrin in rat podocytes. (C) Immunogold particles for PAR3 localize at the cytoplasmic face of the slit diaphragms. FP, foot process.

Based on the analogy of the slit diaphragms with tight junctions, we hypothesized that the aPKC-PAR complex plays a critical role in podocyte slit diaphragms. Consistent with this hypothesis, staining for aPKC, activated phospho-aPKC, PAR6β, and PAR3 revealed that these proteins are strongly expressed in podocytes surrounding the glomerular capillary tufts (Figure 1A) and that they co-localize with nephrin (Figure 1B). Electron microscopy revealed that PAR3 localizes at the cytoplasmic face of the slit diaphragms (Figure 1C). These observations suggest a role of aPKC–PAR complex in the maintenance of structure and function of slit diaphragms.

### Podocyte-specific aPKCλ resulted in focal segmental glomerulosclerosis with disassembly of slit diaphragms

**Figure 2:**
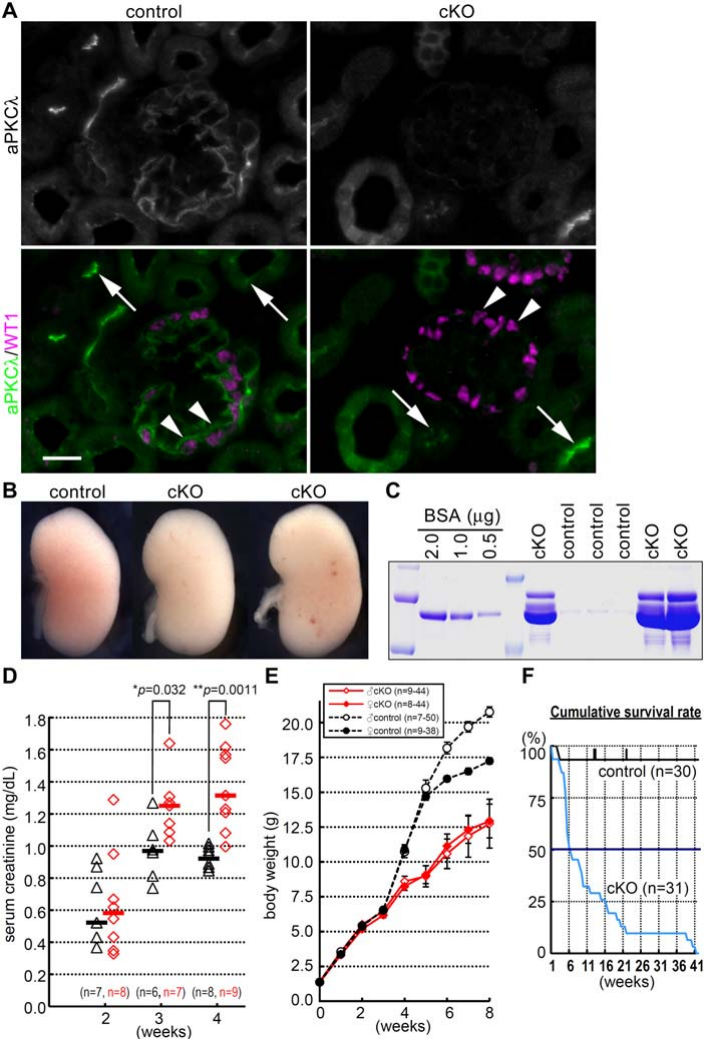
Selective depletion of aPKCλ from mouse podocytes results in renal failure. (A) Double immunofluorescence for aPKCλ (green) and WT1 (magenta) shows that the signals for aPKCλ are below the detectable level in mutant (*aPkcλ* ^ΔE5/floxE5^;*Nphs1-Cre*, cKO) podocytes at P0 (arrowheads), whereas the tubular epithelial cells retained aPKCλ in the mutant (arrows). Bar, 20 µm. (B) No significant difference in the gross appearance of mutant (cKO) and control kidneys at P0. (C) One microliter of urine from each mouse at P0 was analyzed along with bovine serum albumin (BSA) by SDS-PAGE and CBB staining. (D) Serum creatinine concentration in mutant mice compared with controls. Triangles, controls carrying the *Nphs1-Cre* transgene; diamonds, mutants; bars, medians. The *p* values were determined by the two-tailed Mann–Whitney *U*-test. (E) Mutant mice (cKO) show growth retardation by the age of 4 weeks. Values are mean±S.E.M. (F) Kaplan–Meier survival curve for mutant (cKO) and control mice.

To determine whether the aPKC–PAR complex is critical for slit diaphragms, we deleted aPKCλ selectively from podocytes in mice. We deleted exon 5 of the loxP-flanked aPKCλ gene (*Prkci* – Mouse Genome Informatics) using the Cre transgene driven by the podocyte-specific nephrin promoter [18], [19] (*aPkcλ* ^ΔE5/floxE5^; *Nphs1-Cre* ^Tg^ mutant mice) (Supplemental Figure S1). Selective loss of aPKCλ protein in the podocytes at postnatal day 0 (P0) was confirmed by double immunofluorescence for aPKCλ and WT1, a podocyte-specific transcription factor [20] (Figure 2A). Mice of all genotypes were born at the expected Mendelian frequency (data not shown). Although the gross appearance of mutant kidneys was not significantly different from that of control kidneys at P0 (Figure 2B), the mutant mice exhibited nephrotic-level proteinuria on P0 (Figure 2C) and throughout their lives (data not shown). By 3 weeks of age, mutant mice developed severe renal dysfunction, including a significantly increased serum creatinine level and blood urea nitrogen level compared with control mice (Figure 2D and not shown). By 4 weeks of age, mutant mice showed growth retardation (Figure 2E), resulting in a drastically reduced life span with a median age of death of 6 weeks (Figure 2F). These observations indicate that aPKCλ plays a critical role in podocytes in supporting appropriate renal function.

**Figure 3:**
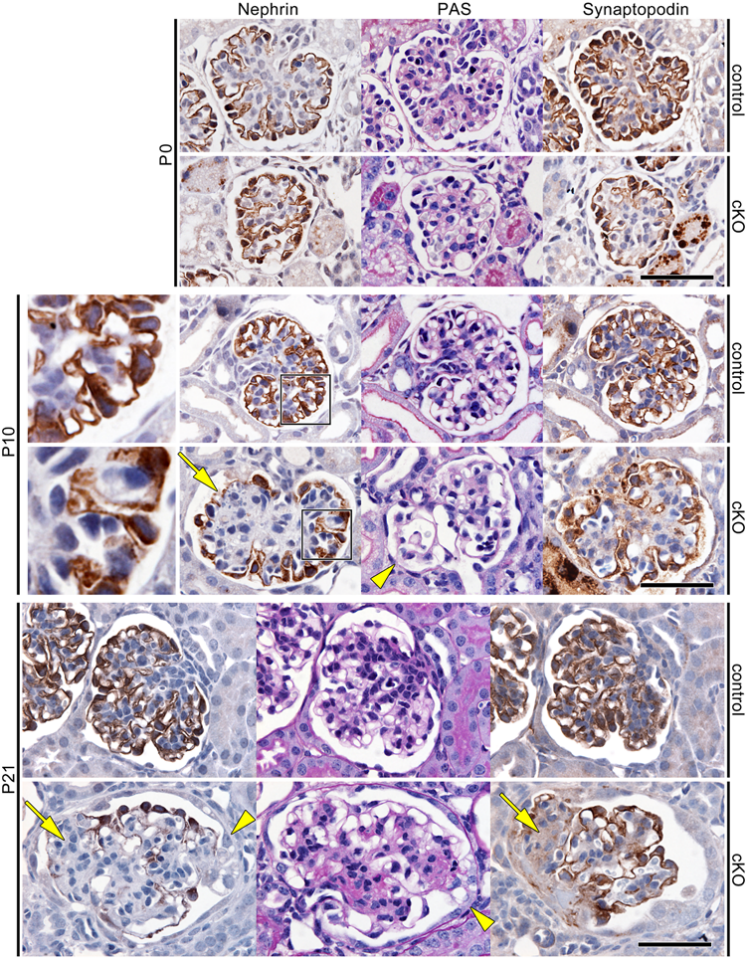
Selective depletion of aPKCλ from mouse podocytes causes FSGS. Periodic acid-Schiff staining (PAS) and immunohistochemistry for nephrin and synaptopodin in mutant (cKO) and control kidneys at P0, P10 and P21 show that mutant mice develop segmental to global glomerulosclerosis. Boxed regions are enlarged to show an irregular pattern of nephrin staining in mutant podocytes. Loss of podocytes (arrows) and occasional adhesion of glomeruli to Bowman's capsules (arrowheads) reveal the development of focal segmental glomerulosclerosis. Consistent with massive proteinuria in mutant mice at birth, PAS staining reveals occasional hyaline droplets, representing reabsorbed urinary protein, in the proximal renal tubules at P0. Bars, 50 µm.

Histopathologic analyses of the mutant kidneys revealed the development of progressive glomerulosclerosis (Figure 3). At P0, the mutant kidneys showed normal nephrogenesis and glomerulogenesis with normal staining patterns of nephrin and synaptopodin [21], an actin-associating protein, in podocyte foot processes. At P10, however, the mutant glomeruli demonstrated partial detachment of podocytes, adhesion of glomeruli to Bowman's capsules, mesangial expansion, dilated capillaries, and an irregular pattern of nephrin staining. At P21, the mutant glomeruli demonstrated more advanced glomerular damage with characteristic segmental to global sclerotic lesions. These histopathologic observations are consistent with the development of severe renal dysfunction in mutant mice by the age of 3 weeks. Together, these are features characteristic of focal segmental glomerulosclerosis.

**Figure 4:**
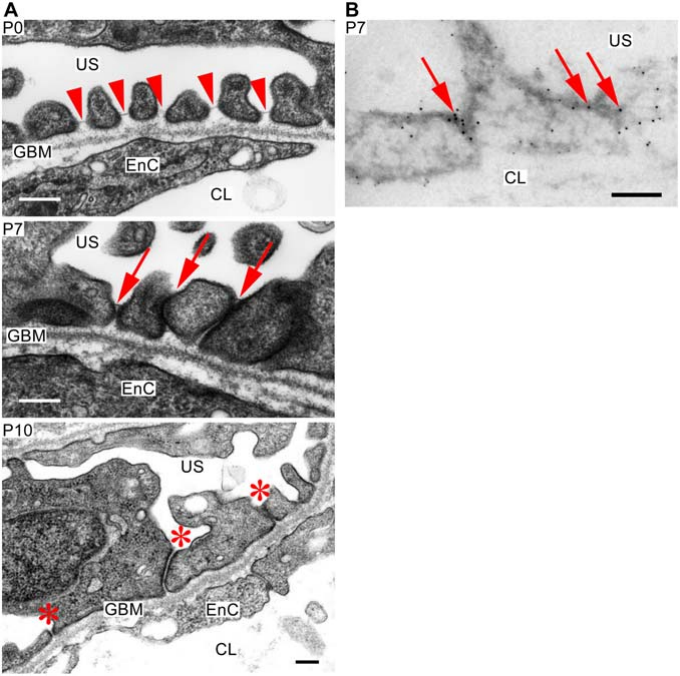
Ultrastructure of mutant podocytes. (A) Although fine foot processes and slit diaphragms are observed at P0 (arrowheads), the foot processes form irregular adhesions and slit diaphragms are apically mislocalized at P7 (arrows). Mutant podocytes at P10 demonstrates effacement of foot processes and irregular adhesions between foot processes (asteriscs). Apparently, these adhesions did not form tight junctions. The glomerular basement membrane (GBM) is not significantly affected. US, urinary space; EnC, endothelial cell; CL, capillary lumen. Bars, 200 nm. (B) Immunogold electron microscopy in mutant podocytes at P7 reveals that podocalyxin (5-nm gold particles) loses its apically restricted localization, whereas ZO-1 (10-nm gold particles) localizes at the cell–cell junctions of podocytes (arrows). US, urinary space; CL, capillary lumen. Bar, 200 nm.

To further analyze the onset of the glomerular damages, we examined the mutant podocytes at an ultrastructural level (Figure 4A). At P0, fine foot processes were embedded in the glomerular basement membrane, and in particular, normal slit diaphragms were formed between the foot processes. These observations, however, do not exclude the possibility that aPKCλ is required for the development of slit diaphragms, because the activation of the nephrin promoter is concomitant with the development of slit diaphragms [22] and *Nphs1-Cre*-mediated recombination cannot be accomplished before the onset of slit diaphragm formation (data not shown). At P7–10, however, we observed apically dislocated slit diaphragms and foot process effacement. Moreover, podocalyxin, a marker protein of the apical domains of podocytes [23], localized not only in the apical but also in the basal domains, whereas ZO-1 localized at the irregular cell–cell junctions (Figure 4B), indicating disturbance in the apico-basal cell polarity. Altogether, these observations indicate that aPKCλ is necessary for the maintenance of slit diaphragm integrity and apico-basal cell polarity in podocytes. This implies that the disturbance in apico-basal cell polarity in podocytes can cause disturbance of the slit diaphragms resulting in glomerulosclerosis.

### aPKC–PAR3 complex associates with the nephrin–podocin complex

**Figure 5:**
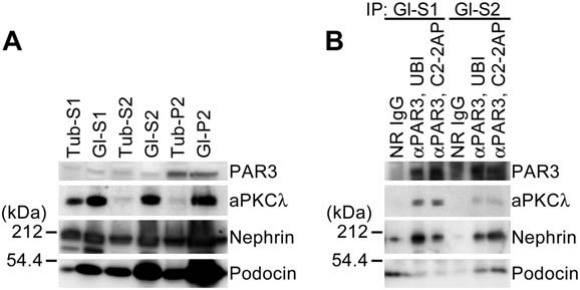
PAR3 forms a complex with aPKC, nephrin, and podocin *in vivo*. (A) The distribution of PAR3, aPKCλ, nephrin, and podocin was analyzed in differentially extracted fractions of rat renal tubules or glomeruli (Tub, Gl). Tub-S1, Gl-S1, highly soluble fractions extracted with 0.1% TritonX-100; Tub-S2, Gl-S2, relatively soluble fractions extracted with 1.0% TritonX-100 and 20 mM CHAPS; Tub-P2, Gl-P2, insoluble fractions. The blotted filter with anti-nephrin antibody was stripped and reprobed with anti-PAR3 antibody. (B) Immunoprecipitation for PAR3 was carried out on both glomerular fractions (Gl-S1, Gl-S2) using two independent anti-PAR3 antibodies (UBI, C2-2AP) and the immunoprecipitates were analyzed by immunoblotting. The blotted filter with anti-nephrin antibody was stripped and reprobed with anti-PAR3 antibody.

To further explore the molecular mechanisms, by which the aPKC–PAR complex organizes slit diaphragms, we examined the potential association between the aPKC–PAR complex and nephrin in podocytes. First, isolated rat renal tubules (Tub) and glomeruli (Gl) were extracted sequentially under different conditions in detergents (see Methods), and analyzed the distribution of each protein by immunoblotting (Figure 5A). Consistent with previous reports [14], [24], [25], nephrin and podocin were concentrated in the Gl–S2 and Gl–P2 fractions, which contain proteins associated with lipid raft microdomains and actin cytoskeletons, respectively. Similarly, aPKCλ from glomeruli, but not from renal tubules, was efficiently concentrated to the Gl–S2 and Gl–P2 fractions, suggesting that a significant amount of aPKCλ in glomeruli codistributes with nephrin and podocin. Next, we used immunoprecipitation to examine whether endogenous PAR3 forms a protein complex with nephrin and podocin. As demonstrated previously [26], aPKCλ was reproducibly coprecipitated with PAR3 from both of the soluble fractions extracted from glomeruli (Figure 5B). Furthermore, nephrin coprecipitated with PAR3 from both fractions. Importantly, podocin consistently coprecipitated with PAR3 from the Gl–S2 fraction. These data indicate that PAR3 forms a complex not only with aPKCλ but also with the nephrin–podocin complex *in vivo*. This suggests that PAR3 can serve as a scaffold to associate aPKC and the nephrin–podocin complex to organize slit diaphragms.

**Figure 6:**
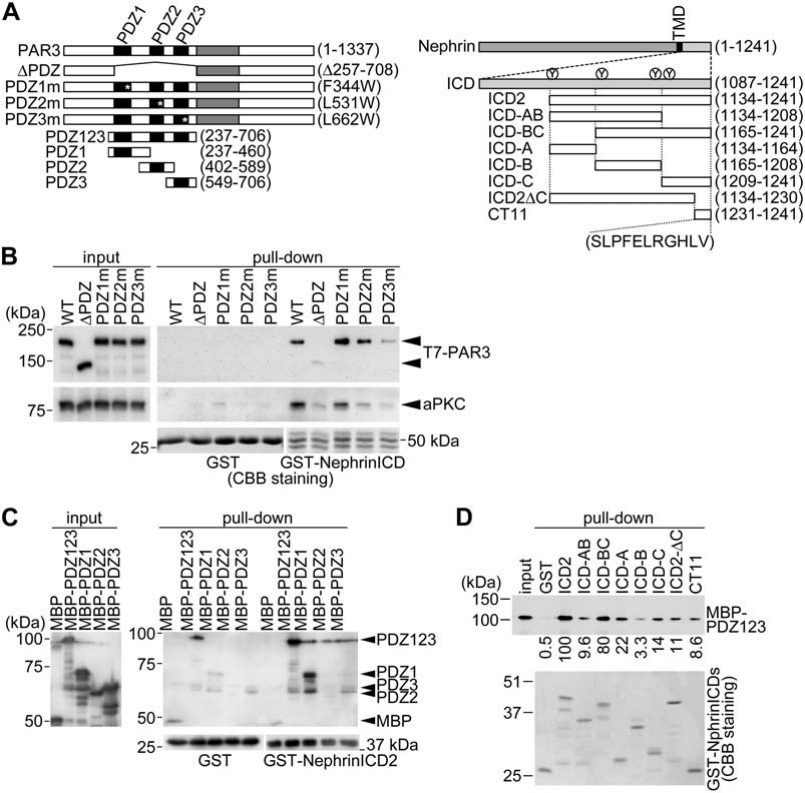
PAR3 binds directly to nephrin *in vitro*. (A) Domain structure, deletion constructs, and point mutants of PAR3 (left) and nephrin (right). The numbers refer to amino acids. PDZ, PDZ domain; aPKC-BR, aPKC-binding region; ECD, extracellular domain; TMD, transmembrane domain; ICD, intracellular domain. The C-terminal amino acids of nephrin show similarities to the type II PDZ-binding motif. The four Ys on nephrin represent the tyrosine residues responsible for binding to phosphatidylinositol-3 kinase p85 or Nck. (B) Mapping of the region in PAR3 that is required for binding to nephrin. Glutathione-*S*-transferase (GST)–nephrinICD-immobilized beads were incubated with 293T cell lysate expressing various T7-tagged PAR3 constructs as indicated. The bound proteins were analyzed by immunoblotting and CBB staining. (C) PAR3 directly binds to nephrin through the PDZ domains of PAR3. Purified GST–nephrin ICD2 was incubated with various PAR3 constructs fused with maltose-binding protein (MBP). The resulting complexes were immobilized on glutathione-Sepharose beads and analyzed by immunoblotting. (D) Mapping of the region in nephrin that is required for binding to the PDZ domains of PAR3. Purified MBP–PDZ123 was incubated with various nephrin constructs fused with GST, each at a final concentration of 50 nM. The resulting complexes were analyzed by immunoblotting and CBB staining. Numbers show relative intensities of the bands.

To examine the region of PAR3 that is responsible for the formation of the complex with nephrin, various PAR3 mutants (Figure 6A) were incubated with immobilized glutathione *S*-transferase fused with the cytoplasmic tail of nephrin (GST–nephrinICD). T7-tagged PAR3 mutants overexpressed in 293T cells specifically interacted with GST–nephrinICD, except for the mutants lacking all PDZ domains or mutated in the third PDZ domain [27], [28], indicating the third PDZ domain is required for the formation of the complex with nephrin (Figure 6B). Endogenous aPKC in 293T cells also interacted with GST–nephrinICD in a PAR3-dependent manner, confirming the formation of a ternary complex of PAR3, aPKC, and nephrin.

We next addressed the possibility of direct interaction between PAR3 and nephrin *in vitro*. Purified GST–nephrinICD2 precipitated the three PDZ domains of PAR3 fused with maltose binding protein (MBP–PDZ123) (Figure 6, A and C). This indicates that the cytoplasmic tail of nephrin can bind directly to the PDZ domains of PAR3. Although GST–nephrinICD2 can bind with the isolated first or third PDZ domains of PAR3, all three PDZ domains are required for efficient binding. To identify the PAR3-binding region in nephrin, we examined the binding ability of various deletion fragments of nephrin (Figure 6A) with immobilized MBP–PDZ123. We found that deletion of the carboxy-terminal 11 amino acids of nephrin (ICD2ΔC) reduced their binding affinity for MBP–PDZ123 and that these 11 amino acids (CT11) can bind to MBP–PDZ123 (Figure 6D). This indicates that CT11 partly mediates the interaction between nephrin with PAR3, and is consistent with the sequence similarity of CT11 to the type II PDZ-binding motif [29], [30]. Furthermore, a region encompassing amino acids 1134-1164 of nephrin (ICD-A) is enough for binding with MBP–PDZ123 (Figure 6D). Altogether, these data suggest that the aPKC–PAR complex interacts with the nephrin–podocin complex by direct binding between the PDZ domains of PAR3 and two cytoplasmic regions of nephrin, including the CT11 and ICD-A regions.

### aPKC activity is required for the appropriate distribution of nephrin and podocin in podocytes

**Figure 7:**
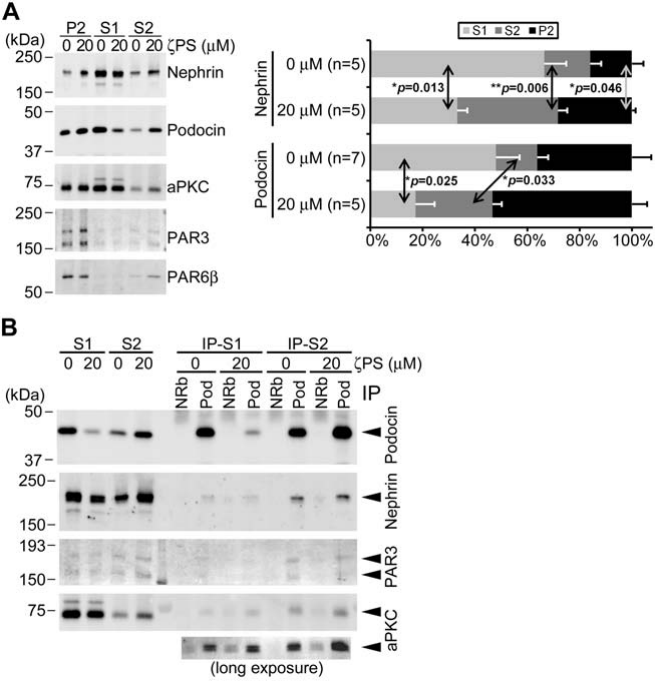
aPKC activity is required for the appropriate distribution of nephrin and podocin. (A) The aPKC inhibitor, myr-ζPS peptide (ζPS), significantly disturbs the appropriate distribution of nephrin and podocin. Differentially extracted proteins (S1, S2, P2; see Methods) from rat glomeruli incubated for 30 minutes with or without 20 µM ζPS were analyzed by immunoblotting (left). Quantitative analysis from five to seven independent experiments is shown (right). Values shown are mean±S.E.M. The *p* values were determined by two-tailed Student's *t*-test. (B) No significant difference in the formation of the nephrin–podocin complex by aPKC inhibitor, myr-ζPS peptide (ζPS). Glomeruli isolated from rat kidneys were incubated for 30 minutes with or without 20 µM of ζPS. The differentially extracted proteins (S1, S2) were incubated with normal rabbit IgG (NRb) or anti-podocin antibody (Pod). Immunoprecipitates (IP-S1, IP-S2) were analyzed by immunoblotting. Numbers show relative intensities of the bands. PAR3 and aPKC were consistently co-immunoprecipitated with podocin irrespective of ζPS treatment.

To determine whether aPKC activity affects the distribution of nephrin and podocin, we treated the isolated glomeruli with or without an aPKC-specific inhibitor [31], [32], and analyzed the distribution of nephrin and podocin in sequentially extracted fractions using 0.1% TritonX-100 (S1) and RIPA buffer (S2) (Figure 7A). Inhibition of aPKC significantly decreased the amount of nephrin in the highly soluble fraction (S1) within 30 min. Contrastingly, the amount of nephrin was significantly increased in the RIPA-soluble (S2) and -insoluble (P2) fractions. The distribution of podocin in the S1 and S2 fractions was similar to that of nephrin. These data suggest that the inhibition of aPKC augments the association of nephrin and podocin with the lipid raft microdomains and actin cytoskeletons.

We further assessed whether the inhibition of aPKC affects the formation of the nephrin–podocin complex in rat glomeruli (Figure 7B). Immunoprecipitation of podocin from the S1 and S2 fractions of the control glomeruli revealed that nephrin was more effectively coprecipitated from the S2 fraction as compared to the S1 fraction. This is consistent with previous reports showing that the functional nephrin–podocin complex is formed in the lipid raft microdomains [25], [33]. Although aPKC inhibition significantly increased the amount of both nephrin and podocin in the S2 fraction, the amount of coprecipitated nephrin was hardly increased. These data suggest that aPKC activity is required in order to prevent the excessive accumulation of nephrin and podocin, which do not form a complex in the lipid raft microdomains. The excessive accumulation of nephrin in the RIPA-insoluble (P2) fraction might lead to the formation of abnormal aggregations of nephrin, which in turn might strongly associate with actin cytoskeletons, because the forced aggregation of nephrin is reported to induce localized actin polymerization through direct binding to Nck [34], [35].

## Discussion

Podocytes are highly polarized cells with apical, basal, and slit diaphragm domains [3], [4]. Because each membrane domain plays a critical role in the selective filtration barrier, and organizing the glomerular basement membrane so that foot processes can anchor in, the regulation of cell polarity is considered to be important for podocytes [1], [3], [4]. We have provided direct evidence that the cell polarity protein aPKCλ is critical for the maintenance of the slit diaphragms. Our data suggest that the aPKC–PAR complex establishes apico-basal cell polarity in podocytes and appropriately arranges the slit diaphragms to form the filtration barrier. Our data further suggest that the association of the aPKC–PAR3 complex with the nephrin–podocin complex regulates the state of equilibrium in which nephrin and podocin are appropriately distributed among raft and non-raft microdomains to avoid unnecessary aggregation of nephrin. Because it is recently reported that PAR3 form a protein complex with Neph1 [36], another nephrin-binding transmembrane protein in slit diaphragms [37], PAR3–Neph1 complex may influence this equilibrium. In the regulation of actin cytoskeletons, the direct binding of the aPKC–PAR3 complex to the cytoplasmic tail of nephrin may compete with Nck and prevent the rearrangement of actin cytoskeletons in podocytes to maintain the integrity of the slit diaphragms [34], [35]. Our observations provide a new pathophysiologic mechanism whereby any condition disturbing aPKC activity can lead to defective organization of membrane domains, damage podocytes, and result in FSGS.

## Materials and Methods

### Materials

cDNA fragments of rat PAR3 and human nephrin were amplified by PCR, subcloned into SRHis/T7, pMAL-c2 (New England Biolabs), or pGEX-6P (Amersham) vectors [26], [27], and sequenced completely. The point mutations in rat PAR3 cDNA were introduced with QuikChange™ Site-Directed Mutagenesis Kit (Stratagene). Human nephrin cDNA was kindly provided by H. Tsukaguchi (University of Tokushima). Rabbit polyclonal anti-PAR3 (Upstate Biotech, Inc. #07-330 and C2-2AP), rabbit polyclonal anti-PAR6β (Beta1-4AP), rat polyclonal anti-PAR6β (BCR12AP), and rabbit polyclonal anti-GST were described previously [26], [27], [38]. The following antibodies were also used: mouse anti-aPKCι (clone 23; BD Transduction Laboratories), rabbit anti-aPKCλ/ζ, rabbit anti-WT1, mouse anti-T7 Omni-probe (Santa Cruz Biotechnology), rabbit anti-phospho-aPKCλ/ζ Thr403/410 (Cell Signaling), anti-MBP (New England Biolabs), guinea pig anti-nephrin, mouse anti-synaptopodin (clone G1D4; Progen), and rabbit anti-Podocin (Sigma).

### Animals

We bred *aPkcλ* ^ΔE5/+^ mice [18] with *Nphs1-Cre* ^Tg^ mice [19] to generate *aPkcλ* ^ΔE5/+^; *Nphs1-Cre* ^Tg^ mice, and bred these mice with homozygous floxed *aPkcλ* (*aPkcλ* ^floxE5/floxE5^) mice [18] to obtain *aPkcλ* ^ΔE5/floxE5^; *Nphs1-Cre* ^Tg^ mutant mice. The *Rosa26R* strain was purchased from the Jackson Laboratory. Serum creatinine levels were measured using a Jaffé assay kit (Wako Pure Chemical) according to the manufacturer's instructions. Male Wistar rats (220–240 g, 7 weeks old) were purchased from Charles River Japan. All animal experimentations were conducted in accordance with the Guidelines for Proper Conduct of Animal Experiments (Science Council of Japan), and all protocols were approved by our institutional review boards.

### Histology and immunostaining

Two-micrometer-thick paraffin sections of 4% PFA-fixed kidney were stained with periodic acid-Schiff (PAS) and hematoxylin (Muto Chemical). For Immunostaining, the sections were autoclaved in target retrieval solution (Dako S3308 or Dako S1700, for 30 min at 90°C and for 10 min at 121°C, respectively), and processed for immunostaining as described previously [38]. The sections were examined and photographed with a DMR microscope (Leica) equipped with a Pro600ES color CCD camera (Pixera) or with a BX50 epifluorescence microscope (Olympus) equipped with a SenSys CCD camera (Photometrics). All images were arranged and labeled using Photoshop 5.5 (Adobe Systems).

### Transmission electron microscopy

The mice were perfused with 2.5% glutaraldehyde in 0.1 M sodium phosphate buffer at pH 7.4 (PB). The kidneys were removed, cut into small pieces, and immersed in 2.5% glutaraldehyde containing 1% tannic acid in 0.1 M PB for 2 h at 4°C. They were then post-fixed with 1% OsO_4_, dehydrated and embedded in epoxy resin. Ultrathin sections were stained with uranyl acetate and lead citrate and then examined under a JEM 1230 electron microscope (JEOL).

### Immunogold labeling

Mouse kidneys were perfused with PLP fixative and immersed in the same fixative for 30 min at 4°C. The samples were rinsed with 5% sucrose for 30 min at 4°C. Tissue samples were then infiltrated with 40% polyvinylpyrrolidone/2.3 M sucrose buffered with 0.1 M PB, embedded on nails, and frozen quickly in liquid nitrogen. Ultrathin sections were cut with an Ultracut UCT equipped with an EM FCS cryoattachment (Leica) at −110°C. Sections were transferred to Formvar-coated nickel grids (150 mesh). Subsequent incubation steps were performed by floating the grids on droplets of the filtered solution. Free aldehyde groups were quenched with PBS–0.01 M glycine, and the sections were incubated overnight with PBS containing 20% fetal bovine serum (FBS). Next, the grids were incubated with affinity-purified rabbit anti-podocalyxin antibody (1∶200 dilution with PBS containing 20% FBS) and mouse anti-ZO-1 antibody (Zymed, 1∶100 dilution) for overnight at 4°C. The grids were then incubated with anti-rabbit IgG coupled with 5 nm-gold (diluted 1∶100) and anti-mouse IgG coupled with 10 nm-gold (diluted 1∶100) for 1 h. After immunostaining, the samples were fixed with 2.5% glutaraldehyde buffered with 0.1 M PB (pH 7.4). The sections were then contrasted with 2% uranyl acetate solution for 20 min, and absorption-stained with 3% polyvinyl alcohol containing 0.2% uranyl acetate for 20 min. All sections were observed using a JEM 1230 electron microscope.

### Sequential extraction of glomeruli and immunoprecipitation

Rat kidneys were perfused with ice-cold HBSS(+) containing protease inhibitors (2 mg/L each of antipain, leupeptin, aprotinin, and pepstatin A; 2 mM benzamidine; 1 mM PMSF) under Nembutal anesthesia, and renal tubules and glomeruli were isolated by graded sieving at 4°C in the presence of protease inhibitors [12]. The isolated glomeruli were incubated with or without a membrane permeable myristoylated pseudosubstrate peptide inhibitor specific for atypical PKC subtypes [31], [32] (myr-SIYRRGARRWRKL, myr-ζPS peptide; Peptide Institute, Inc.). Each tissue was extracted with IP buffer (20 mM HEPES–NaOH (pH 7.5), 150 mM NaCl, 50 mM NaF, 25 mM β-glycerophosphate, 10% glycerol, 1 mM DTT, 1 mM EDTA, protease inhibitor cocktail (Sigma)) containing 0.1% TritonX-100 (Tub-S1 and Gl-S1 fractions). The insoluble fractions were precipitated by centrifugation (20,000 *g*, 30 min, 4°C), further extracted with IP buffer containing 1% TritonX-100 and 20 mM CHAPS with (RIPA buffer) or without 0.1% SDS (Tub-S2 and Gl-S2 fractions, which include proteins associated with lipid raft microdomains). The insoluble fractions (Tub-P2 and Gl-P2, which include proteins tightly associated with the cytoskeletons) were precipitated by centrifugation (20,000 *g*, 30 min, 4°C). Each soluble fraction was incubated with either of the antibodies indicated and the precipitated complexes were analyzed by immunoblotting. The signals were captured with an LAS-3000mini luminoimage analyzer (Fujifilm) and quantified using MultiGauge software (Fujifilm).

### GST pull-down assay

Purified GST fusion proteins were separately incubated for 2 h at 4°C with 293T cells expressing the PAR3 proteins extracted with 25 mM Tris-HCl (pH 8.0), 100 mM NaCl, 5 mM EDTA, 1% TritonX-100, and protease inhibitor cocktail (Sigma), or with purified MBP-fusion proteins in 20 mM HEPES–NaOH (pH 7.5), 100 mM NaCl, 5 mM EDTA, 1% TritonX-100, 0.5 mg/ml bovine serum albumin, and 10% glycerol. The GST fusion proteins were collected with glutathione–Sepharose beads (Amersham) and analyzed by immunoblotting.

### Statistical analysis

The two-tailed Mann–Whitney *U*-test (VassarStats, http://faculty.vassar.edu/lowry/VassarStats.html) or the two-tailed Student's *t*-test (Microsoft Excel 2007) were used to analyze the differences between the pairs of groups. Values were regarded significant at *p*<0.05.

## Supporting Information

Figure S1 The *Nphs1-Cre* transgene mediates podocyte-specific recombination. (A)Podocyte-specific recombination of loxP-flanked regions mediated by the *Nphs1-Cre* transgene. Red triangles represent loxP sequences. (B)Specific Cre activity restricted to the glomeruli was confirmed in the kidneys of Rosa26R reporter mice carrying the *Nphs1-Cre* transgene. X-gal staining of the *Rosa26R*;*Nphs1-Cre* kidney indicates the glomerular-specific β-galactosidase expression caused by the *Nphs1-Cre* transgene. Bars, 200 µm.
(2.63 MB TIF)
