# Supplementary figures and images for: An Essential Role of the Universal Polarity Protein, aPKCλ, on the Maintenance of Podocyte Slit Diaphragms
Source: PLoS One. 2009 Jan 14;4(1):e4194. doi: 10.1371/journal.pone.0004194 (PMC2614475; doi:10.1371/journal.pone.0004194)

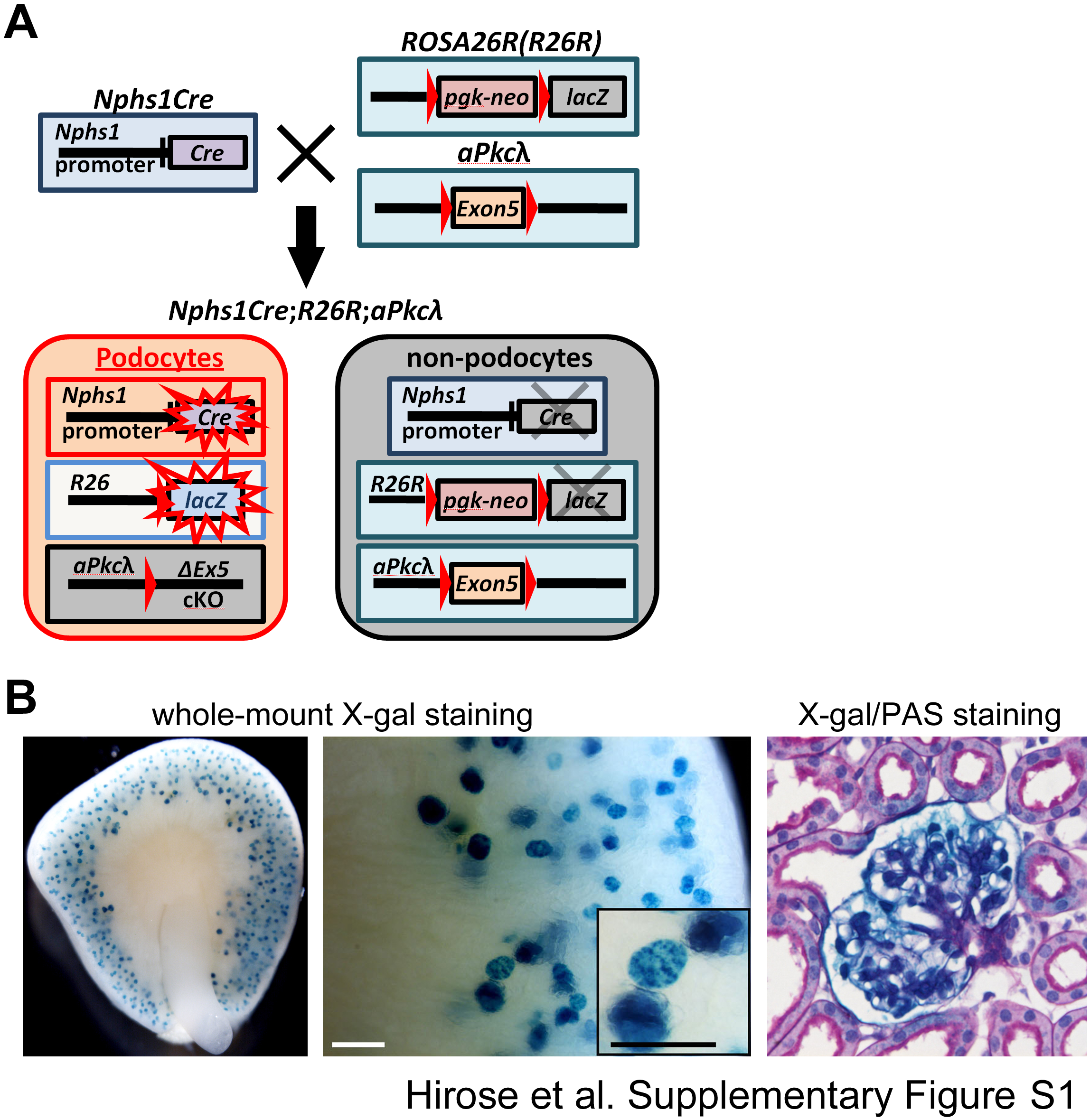

Supplement: Figure S1 — The Nphs1-Cre transgene mediates podocyte-specific recombination. (A)Podocyte-specific recombination of loxP-flanked regions mediated by the Nphs1-Cre transgene. Red triangles represent loxP sequences. (B)Specific Cre activity restricted to the glomeruli was confirmed in the kidneys of Rosa26R reporter mice carrying the Nphs1-Cre transgene. X-gal staining of the Rosa26R;Nphs1-Cre kidney indicates the glomerular-specific β-galactosidase expression caused by the Nphs1-Cre transgene. Bars, 200 µm. (2.63 MB TIF) [file pone.0004194.s001.tif]
